# Neural network based classification of acute toxicity of phthalate esters to fathead minnow

**DOI:** 10.1186/1758-2946-5-S1-P47

**Published:** 2013-03-22

**Authors:** V Arulmozhi, Rajesh Reghunadhan

**Affiliations:** 1 Dept. of Computer Applications, Tirupur Kumaran College for Women, India; 2 Dept. of Computer Applications, Bharathiar University, Coimbatore - 641046, India

## 

Chemoinformatics, the brain child of Frank Brown [1], has emerged as new branch of science by the technological marriage of information technology and chemistry [2,3]. Quantitative Structure-Activity Relationships (QSARs), needed for the analysis/drug-design, are in the form of structural alerts that incorporate molecular substructures, presence/absence of activity, structural relationships, etc. These QSAR can be used to perform an initial screening for classification/labelling.

QSAR of acute toxicity (from ECOSAR/TOPKAT) is used to classify phthalates into toxic/nontoxic group. The multidimensional features obtained from QSAR are having high overlapping and it is very difficult to classify it into toxic/nontoxic groups. This paper presents the classification of acute toxicity of phthalate esters to fish (Fathead Minnow) with QSAR features using Neural Network. The dataset consists of features for 324 chemicals and are available in the technical report by Tatiana Netzeva and Andrew Worth [4]. We have used four features for classification purpose namely, MW (molecular weight), WSol (water solubility), K_ow _(octanol-water partition coefficient), LC (lethal concentration). The toxicity values are divided into four categories, namely, no concern, harmful, toxic, and very toxic.

Neural network [5] with 100 hidden neurons is trained using scaled conjugate gradient algorithm for about 2000 epochs. The following code shows part of the Matlab code which is used in our classification.

net=newpr(traininst',trainlabels',no_hidden_neurons);

net.trainParam.epochs = 2000; net.trainParam.goal = 0.00001;

net.trainParam.min_grad = 0.000000000000000000000001;

net.divideParam.trainRatio = 1; net.divideParam.valRatio = 0;

net.divideParam.testRatio = 0; net=train(net,traininst',trainlabels');

output=sim(net,testinst'); [err,cm]=confusion(testlabels',output);

successrate=sum(diag(cm))/sum(cm(:))

The classification performance is promising. The classification rate after two cross validation simulated over 100 runs is 91.36%.
